# What Are the Characteristics of User Texts and Behaviors in Chinese Depression Posts?

**DOI:** 10.3390/ijerph19106129

**Published:** 2022-05-18

**Authors:** Jingfang Liu, Mengshi Shi

**Affiliations:** School of Management, Shanghai University, Shanghai 201800, China; jingfangliu@shu.edu.cn

**Keywords:** online social media, depression, natural language processing, text analysis

## Abstract

Social media platforms provide unique insights into mental health issues, but a large number of related studies have focused on English text information. The purpose of this paper is to identify the posting content and posting behaviors of users with depression on Chinese social media. These clues may suggest signs of depression. We created two data sets consisting of 130 users with diagnosed depression and 320 other users that were randomly selected. By comparing and analyzing the two data sets, we can observe more closely how users reveal their signs of depression on Chinese social platforms. The results show that the distribution of some Chinese speech users with depression is significantly different from that of other users. Emotional sadness, fear and disgust are more common in the depression class. For personal pronouns, negative words and interrogative words, there are also great differences between the two data sets. Using topic modeling, we found that patients mainly discussed seven topics: negative emotion fluctuation, disease treatment and somatic responses, sleep disorders, sense of worthlessness, suicidal extreme behavior, seeking emotional support and interpersonal communication. The depression class post negative polarity posts much more frequently than other users. The frequency and characteristics of posts also reveal certain characteristics, such as sleep problems and reduced self-disclosure. In this study, we used Chinese microblog data to conduct a detailed analysis of the users showing depression signs, which helps to identify more patients with depression. At the same time, the study can provide a further theoretical basis for cross-cultural research of different language groups in the field of psychology.

## 1. Introduction

Depression is one of the most common mental health problems, and it is estimated that more than 300 million people worldwide suffer from depression [1]. Long-term mild or moderate depression will affect the quality of life of patients and their family members, and severe depression patients will even engage in extreme risk behaviors such as suicide. Fortunately, several studies have shown that early identification and treatment of depression can ameliorate the negative effects of the disorder [2,3,4]. Although there are psychotherapies, drug therapies, and other ways to treat depression, due to the stigma of the disease, most patients will not go to medical institutions because of psychological or mental problems. They often choose to go to the hospital only when they have physical discomfort symptoms, so the early detection and treatment rate of depression is low [5,6,7].

Recently, there has been increasing interest in using social media to identify, prevent or intervene in different types of mental disorders [8,9,10,11]. Lyons et al. [8] compared the language differences of users with different forms of mental distress (generalized anxiety disorder, borderline personality disorder, severe depression, obsessive-compulsive disorder, and schizophrenia) in online communities and found that users who experienced any form of pain used singular personal pronouns more frequently and had more negative emotions. There is a high degree of similarity among patients with different forms of mental distress. At the same time, existing studies have shown that there are certain changes in the mode of mental illness patients’ participation in online social media. Vedula and Parthasarathy [9] found that depressed users tend to gather with other potential depressed users in their social network.

In early studies focusing on depression, most of them were aimed at countries whose mother tongue was English, and the research platforms were mainly Twitter [12,13,14,15] and Facebook [16,17], while there is less discussion on the content generated by patients with depression on Chinese social platforms. However, there are differences in the expression of depression in different cultural backgrounds. Tsugawa et al. [18] found that certain linguistic characteristics of patients exist in both English and Japanese tweets, indicating similar cross-cultural tendencies, but Asians are more cautious than Americans in self-disclosure [19]. The use of language can only highlight its true meaning based on its cultural background. There are obvious differences between Chinese and English in expression characteristics, language style, and expression methods, such as differences in tenses in English. In addition, social and family environments are the key factors leading to depression, and patients with depression may also have different ways of participating in social networks and discussing topics under different social and cultural backgrounds. Ramírez-Esparza et al. [20] analyzed depression posts in English and Spanish and found that depression patients who wrote in Spanish were more likely to mention relationship problems than those who wrote in English, and English patients were more likely to mention medical problems. We chose Weibo [21], the most widely used social media platform by Chinese users, to identify relevant signals in text content and posting behaviors of patients with depression. In this work, we try to answer the following questions: (1) Based on Chinese morphology, what is the unique linguistic style of the users showing depression signs? (2) Under the Chinese social media platform, what is the emotional distribution and inner world of patients? (3) Compared with normal users, what is the post behavior pattern of depression class when participating in social networks?

## 2. Materials and Methods

### 2.1. Data Collection

As social media such as Twitter and Facebook are blocked in China, Weibo is one of the most widely used real-time online social media platforms in China [22]. The functions of Weibo and Twitter are basically the same, but there are still significant differences in user behaviors on the platform due to cultural differences [21]. The openness and anonymity of Weibo enable users to build relationships with different strangers, freely and securely express themselves, and even discuss some private issues. At present, its monthly active users have reached 573 million, and its data have been widely used in research on medical topics, such as organ donation [23], mental disorders [24], AIDS [25], and so on.

Users can choose to join interested communities, share, and discuss topics on the Weibo platform. Taking the Weibo Super Topic of “Depression” in the medical and health module as an example, as of January 2021, the number of fans had reached 270,000, and the number of posts posted was 653,000, ranking first in the medical and health community. We preliminarily screen users with depression tendencies in the depression community and analyze all posts on the user’s personal interface to determine whether they are suffering from depression. The specific criteria are the depression diagnosis certificate and the text or picture information of depression-related drugs posted by the user, which can exclude some nondepressed users who pretend to have depression to gain sympathy from others or other purposes. A total of 170 user IDs with depression were selected. We randomly selected 400 other Weibo users, but it was not completely ruled out that they had a history of depression or suffered from other diseases. According to the ID of each user, we spent three months to capture the timeline data of these users at one time, including the user’s posting content, posting time, shared pictures, location, and other information. A total of 396,152 Weibo data points were obtained, including 70,550 posts on depression and 325,602 other posts.

To ensure the accuracy of the subsequent analysis and the high reliability of the results, we filtered the noise data. First, we eliminated nonpersonal accounts, including marketing accounts, official accounts, community managers, etc. At the same time, we deleted users with fewer than 60 original posts. Second, according to the content published by the users showing depression signs, we estimated the approximate time of their illness. We deleted the content published before their illness to reduce the interference of noisy data. After strict screening and data filtering, the data set includes 130 depression samples and 320 other samples, and the numbers of Weibo posts are 42,827 and 293,102, respectively. The raw data obtained include not only the text information shared by the user but also other meaningless field information, such as hashtags, emoticons, positioning information, shared pictures, and videos. Therefore, before data analysis, we adopted a regular matching method to preprocess the original data. The final data information is shown in Table 1.

### 2.2. Data Analysis

Our goal is to analyze the differences in text content and posting behavior between depression class and others. Next, we will introduce the selection of each group of features in detail. At the same time, since the number and length of posts vary from user to user, normalization was carried out for each variable, and descriptive statistics were expressed as the mean value and standard deviation. The variables of posting behavior were measured by repeated variance to compare the changes of each index at different times, and the other indexes were compared with Mann–Whitney test to compare the differences between groups. The analysis used IBM SPSS statistics for Windows version 25.0 and *p* < 0.05 was considered significant.

- Text analysis

Part-of-speech analysis: We used the Python third-party library Chinese word segmentation tool Jieba for part-of-speech tagging. According to the speech information of each word, the frequency of 20 part-of-speech categories was counted. See Table 2 for the specific description and examples of Chinese words.

Emotion words: The basic emotion words in each post are identified through the Chinese Affective Lexicon Ontology of Dalian University of Technology [26], which includes seven fine-grained emotion dimensions of happy, like, anger, sad, fear, surprise, and disgust.

Negative words: We chose the list of negative categories in the Chinese sentiment dictionary HowNet [27] to identify negative components.

Interrogative words: Based on Chinese, 16 interrogative pronouns are used to count the question words in the posts.

Personal Pronouns: This paper chose to analyze the first-person singular and plural, the second-person singular and plural, and the third-person singular and plural. In addition, we found that the pronoun ‘别人’ (others) also appeared frequently in the posts of depression class, so we added the analysis of this term.

Polarity analysis: We used Baidu Smart Cloud Platform’s text sentiment analysis API (https://ai.baidu.com/tech/nlp_apply/sentiment_classify (accessed on 15 February 2021)) to mark all original posts. The API returns three emotion labels: negative as 0, neutral as 1, and positive as 2.

Topic analysis: Using unsupervised machine learning technology of latent Dirichlet allocation (LDA) [28] to identify potential topic information.

- Posting behavior analysis

Posting time: The number of posts per user in seven days of a week and the number of posts per user in 24 h of a day were counted.

Posting habits: Posts on Weibo are sourced from original content and reposted by others, while users can choose whether to share pictures or use location functions when posting original posts. Therefore, we discussed the posting habits of different users, mainly including original microblog posting, microblog posting with pictures, and positioning microblog posting.

## 3. Results

- Text Analysis

The nonparametric Mann–Whitney U test results showed that the distribution of 16 parts of speech, such as nouns, verbs, and time words, between depression class and otherswas statistically significant, while there was no significant difference between place words, prepositions, auxiliary words, and modal words. In addition, the statistical analysis results showed that, except for emotional anger and third-person singular, the characteristics among each group were statistically significant. The specific statistical results are shown in Table 3.

- Posting behavior analysis

**Figure 1:**
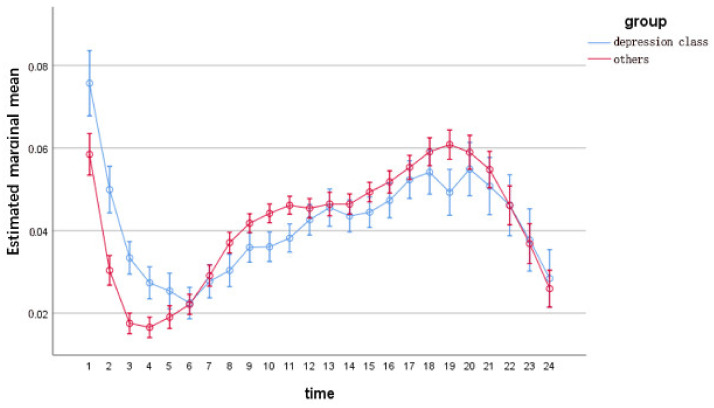
Post rate per hour for depression class and others (mean ± 2 standard error).

**Figure 2:**
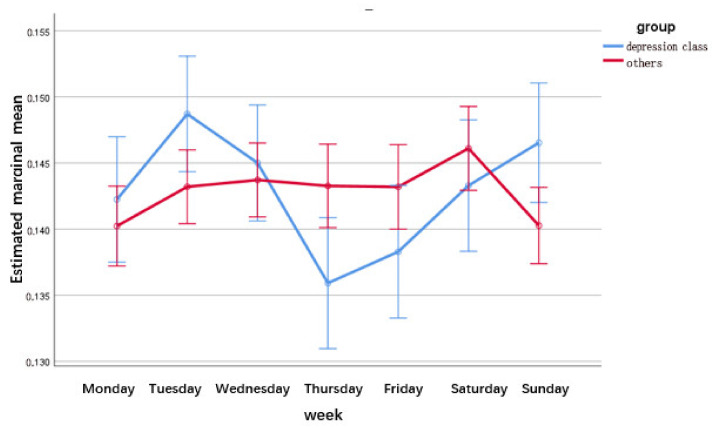
Post rate of depression class and others within a week (mean ± 2 standard error).

Regarding the analysis of posting time, we compared the difference between users’ posting frequency per hour and per week. The statistical result of the frequency of posting by users within 24 h a day was (Greenhouse–Geisser F = 8.374, *p* < 0.001), and the result for seven days a week was (Greenhouse–Geisser F = 6.984, *p* < 0.001). The details are shown in Figure 1 and Figure 2.

Regarding the analysis of posting habits, we chose to compare whether it is an original Weibo post, a Weibo post with pictures, and a positioning Weibo post. Through the independent sample Mann–Whitney U test, significant differences were found in the three posting habits of users with depression and other users. The specific results are shown in Table 4.

## 4. Discussion

- Part of speech

Through part-of-speech tagging and word statistical analysis, users showing depression signs are shown to have higher use of verbs, pronouns, adverbs, conjunctions, set phrases, and idioms than other users, while other users use more nouns, time words, interjections, onomatopoeia, etc. Among them, verbs and adverbs are used more frequently than other users, while nouns, place words, nouns of locality, numerals, quantifiers, etc., are used less frequently, reflecting that patients with depression are less interested in other things and individuals around them [29]. More behavioral words are related to known sensitive disclosures [30], while adverbs are often used to express a strong emotion, which is related to extreme emotional changes in users showing depression signs. At the same time, conjunctions also appear more frequently in the content of posts for depression because patients tend to publish more detailed posts to tell their own experiences [31], so they use more conjunctions to express transitions, cause and effect, and choices. In contrast, the content posted by other patients usually involves all aspects of life, and other part-of-speech morphemes are used relatively more frequently. For example, onomatopoeia is often used in Chinese to describe certain sounds and things, reflecting a certain relaxed mood. However, users showing depression signs less frequently use onomatopoeia, while other users’ posts tend to use such words to vividly express the characteristics of things and the state of actions.

**Figure 3:**
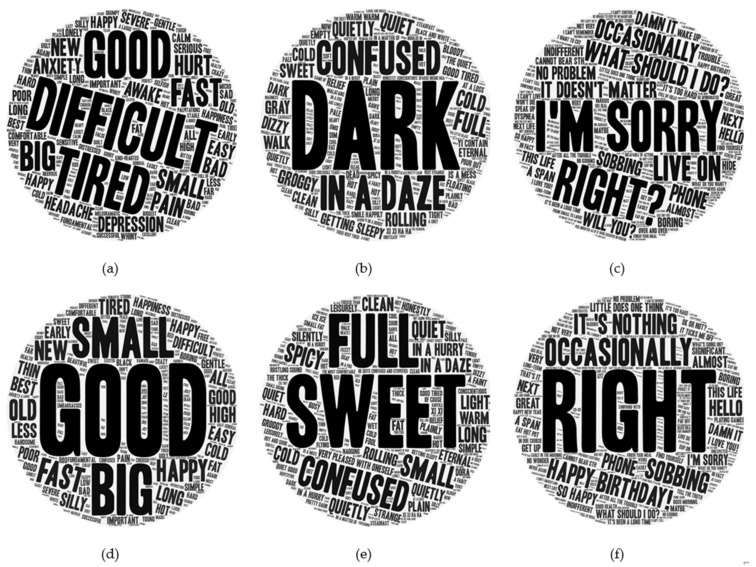
Adjectives, descriptive words, and idiom word clouds: (**a**) The word cloud of adjectives for depression class; (**b**) The word cloud of descriptive words for depression class; (**c**) The word cloud of idioms for depression class; (**d**) The word cloud of adjectives for others; (**e**) The word cloud of descriptive words for others; (**f**) The word cloud of idioms for others.

In addition, we counted the high-frequency words in some parts of speech and found that the nouns used by depression class referred to symptoms of mental illness and treatment-related information, such as mood, medicine, doctor, nausea, sleeping pills, black dog, etc., which had a low frequency or even no occurrence in the control data set. Similarly, some verbs appeared more frequently in the depression dataset, such as crying, fear, leaving, and suicide. Figure 3 shows the word cloud distribution of the two data sets on adjectives, descriptive words, and idioms. Notably, depression class are usually negative in their use of adjectives and descriptive words. Although other users also have negative emotions, users showing depression signs have a strong negative tendency and depressed emotional state. These words reflect their unstable mental state and possible depressive symptoms. Idioms mainly reflect that patients with depression have a strong sense of self-blame, guilt, and uselessness, and excessively belittle themselves, which is consistent with the known psychological state of patients.

- Emotion

The frequency of use of different categories of emotional words is important information in user-generated content, and abnormal emotional preference is one of the important symptoms of depression patients. Although the like affective is the most common in the two types of data sets, there is a significant decrease in the depression data set, and the frequency of using happy emotions is also significantly reduced. At the same time, we observe that the frequency of surprise emotions in the data sets of other users is relatively high, which reflects that depression class may be indifferent to the occurrence of other events around them, and their interest is significantly reduced. In contrast, disgust emotions are used more frequently in the depression class, which is consistent with previous clinical studies. The sensitivity and tendency of disgust are higher than the existence of overall negative emotions [32]. At present, a large number of scholars have found a close correlation between depression and disgust [33,34,35]. Even though anger emotions are more highly expressed in depression data sets, there is no difference in the distribution of anger emotions between the two data sets, which is different from the existing studies [14,36,37]. We speculate that this may be due to the Chinese language characteristics leading to no obvious boundary between users’ expression of disgust and anger emotions. The frequency of sad emotions in the depression data set is almost twice that of other users. The overall expression intensity of the depression data set in the fear emotion is also relatively high. Previous studies have found that the emotion of fear is a factor related to depression [15]. For adolescents, higher fear levels are a significant risk factor for later onset of major depressive disorder [38].

- Personal pronoun

Depression class use more first-person singular pronouns, which is consistent with numerous previous studies [9,39,40], as high self-awareness is a known psychological attribute in psychiatric patients [41]. At the same time, the collective meaning of ‘我们’ (we) is less used, which indicates that the attention of patients is mainly focused on personal problems related to themselves [42].

Regarding the use of second-person singular and plural forms, some divergent results emerged from previous studies [9]. In this study, it is found that ‘你’ and ‘你们’ appear more frequently in the depression data set. We speculate that on the one hand, depression class would post seeking help, support, and advice from others [43], and on the other hand, that the words ‘你’ and ‘你们’ carry an extreme form of exclusion in Chinese expressions, with patients preferring to talk about themselves in an isolated way, and therefore using second-person singular and plural forms more often.

Another interesting finding is that depression class used more third-person plural pronouns and vocabulary ‘别人’ (others), which is significantly different from other users in frequency. Stigmatization of depressive disorders leads to a lack of social identification among patients, who usually remain closely related to their community members [9]. The increased use of these words may reflect patients attributing themselves to that disease group and talking about other groups in an exclusive way. The third-person singular form did not differ between the two data sets. However, more use occurs in patients with borderline personality disorders [8], which are highly correlated with previous painful experiences (e.g., abuse) in this category of patients, and provides a new perspective to explain the differences between different psychiatric disorders.

- Specific words

The results of the analysis of negative words show that, in general, the frequency of negative words in the depression data set is higher. Patients with depression usually have self-denial and negatively treat things around them. Regarding interrogative words (e.g., 什么 (what), 为什么 (why), 哪里 (where)), they appear more frequently in depression data sets. Since our depression dataset is selected in the Weibo depression community, the content of the patients’ posts may involve asking other patients in the community for information related to the disease, and seeking explicit feedback or suggestions from the community [43]. In fact, this phenomenon also reflects the mental state of the users showing depression signs, such as confusion and greater mood swings.

- Polarity

We also explored the polarity of the two data sets, and there was a significant statistical difference between depression class and others in emotional polarity. The most significant point is that the negative polarity of depression class is more severe than that of other users. Indeed, this is not difficult to anticipate because depression class often repeatedly and uncontrollably think about their somatic symptoms, past failures, disease treatment information, or negative experiences [44]. Excessive attention to negative information will trigger a vicious circle of negative emotions, leading to more serious and longer-lasting depression. Furthermore, Seabrook et al. [17] suggest that instability in negative polar expressions on social platforms is an early sign of depression. Positive polarity appeared more often in the control set, and the two data sets had larger gaps. Thus, analyzing polarity propensities in dynamic updates also provides a new opportunity to identify depressed patients.

- Topic Analysis

Topics reveal important information about how people talk about their depression experience on Weibo. We use the widely used theme model LDA to describe the inner world of people showing depression signs. Unlike other social media platforms, Weibo users make extensive use of anonymous information, so they are more likely to share issues that have not been previously discussed with anyone. Table 5 shows the main topics discussed by depression class.

- Posting time

The first is about the frequency of user postings per hour. The main difference is that depression class is most active in the early morning and peaks at 0:00. Internet activities late at night are a known behavioral feature of patients with depression [16]; the underlying reason is that they often have sleep disturbances. Chronic insomnia is a prodromal symptom for the development of major depressive disorder, and in Lustberg’s study, it was shown that eight out of every ten depressed patients experience an exacerbation of symptoms at night [45].

Second, comparing the posting rates of depression class with others in a week, the posting rates of other users fluctuate less in a week and increase significantly on Saturday, while the posting rates of depression class reach their peak and trough on Tuesday and Thursday, respectively. We speculate that the main reason for this phenomenon is that users showing depression signs have more time to socialize and vent their emotions after the end of a week of work and study, so there is a clear upward trend after Friday. Immediately following Monday also implies the beginning of complex interpersonal and life learning, which requires them to take more roles and complete the tasks undertaken.

- Posting habits

Regarding the posting habits of the two types of users, it mainly discusses the posting rate of original Weibo content, whether to post pictures and choose positioning. According to the results, the depression data set contains more original microblogs, and the content shared by other users involves more picture information and geographic location information. The higher posting rate of original Weibo microblogs reflects that patients have fewer reposts of other microblogs than other users, which means that users showing depression signs pay less attention to other external things, and have less desire for social interaction [13]. However, a study on the Facebook platform found that the number of nonoriginal posts in a user’s status update was strongly associated with depressive symptoms, and this association was mediated by neurotic personality [16]. This is contrary to the results of our study. Since Weibo is a Chinese social media platform that consists of a social network built by “strangers” and Facebook’s two-way following mechanism makes users more familiar with each other, depression patients have different levels of willingness to self-disclose on different social media platforms.

There is also an interesting finding with fewer pictures and location information, and these cues often involve more privacy information. The stigma of the disease causes depressed patients to be reluctant to freely express their emotions and opinions [46]. Less personal information can encourage patients to make more self-disclosures without worrying about being identified by others.

## 5. Conclusions

Our research combines Chinese text content and user posting behavior to detect relevant signals of users showing depression signs, and the results are helpful for other scholars to study the related information on social media which is not English. In the Chinese text content, we set out to study the Chinese lexical information, and found that the patients are also significantly different from other users in some specific Chinese parts of speech. Patients also differ in their preferences for expressing emotions and themes in different cultural contexts. There are also differences in patients’ preferences for expressing emotions and themes in different cultural backgrounds. In addition, We found that depressive users’ social media posting behavior was also closely related to their own illness. Due to different mechanisms of attention from other platforms, patients in Weibo communities prefer to update original dynamic content, but also reduce privacy information disclosure.

Importantly, this study has certain limitations, which need to be further addressed in future studies. The diagnosis of depression still requires the judgment of a qualified doctor. Through social media, we cannot fully understand the patient’s other symptoms and the real situation, but our research can provide preliminary judgments for some users who have difficulty determining whether they are sick, and can track severely depressed patients who have dangerous and extreme behaviors. Of course, the premise of all these interventions requires respecting the personal wishes of social media users and supporting the provision of personal privacy. In addition, as depression is occurring in patients at a younger age, most of the users participating in the Weibo depression community are adolescents, so the study sample may not be representative of the general population.

Future research aims to explore language differences and common factors among different types of psychiatric disorders, providing a basis to explain the high rate of concurrence that exists between different disorders. Meanwhile, in addition to different levels of depression being noteworthy, we also recommend targeting different types of depression for research, such as seasonal affective disorder, bipolar disorder, and postpartum depression. Our research combines Chinese text content and user posting behavior to detect depression-related signals, confirming and expanding previous findings on English posts. This study may provide clinicians and caregivers with more clues about the detection and identification of depression by understanding patients’ activities on social media, as a supplement to traditional clinical diagnosis; it may also provide a theoretical basis for cross-cultural research on different language groups in the field of psychology.

## Figures and Tables

**Table 1:** Data set statistics.

Data	Category	User	Post
Candidate	Depression class	170	70,550
	Others	400	325,602
Final dataset	Depression class	130	42,827
	Others	320	293,102

**Table 2:** Part-of-Speech Tagging and Examples.

Tag	Describe	Example
n	Noun	图片 (pictures), 口罩 (masks)
v	Verb	看见 (see), 认识 (recognize)
a	Adjective	小 (small), 美好 (beautiful)
m	Numeral	四 (four), 2020
q	Quantifier	天 (day), 系列 (series)
r	Pronoun	我 (I), 那些 (those)
d	Adverb	非常 (very), 大概 (probably)
u	Auxiliary Word	的 (of), 等等 (etc.)
e	Exclamation	哇塞 (Wow), 嚯 (Ooh)
i	Set Phrase	爱屋及乌 (love me love my dog),不遗余力 (spare no effort)
o	Onomatopoeia	哈哈哈 (ha ha ha), 咕嘟 (gurgle)
t	Time Word	今天 (today), 夏天 (summer)
b	Attributive Word	女 (female), 整个 (whole)
y	Modal Particle	啊 (Ah), 哇 (Wow)
f	Noun of Locality	外面 (outside), 前后 (front and back)
s	Place Word	家里 (home), 路上 (on the road)
z	Descriptive Word	默默地 (silent), 美滋滋 (happy)
p	Preposition	被 (by), 在 (in)
c	Conjunction	如果 (if), 所以 (so)
l	Idiom	对不起 (sorry), 没什么 (nothing)

**Table 3:** Characteristics of user posts’ statistics results.

Category		Depression Class *n* = 130		Others *n* = 320		*p* Value
		Mean (%) ± SD		Mean (%) ± SD		
Part of Speech	Noun	14.72%	0.0220	18.08%	0.0260	***
	Time word	5.79%	0.0194	6.81%	0.0182	***
	Place word	0.46%	0.0016	0.47%	0.0018	0.871
	Noun of locality	1.30%	0.0034	1.51%	0.0035	***
	Verb	29.46%	0.0145	28.26%	0.0148	***
	Adjective	4.83%	0.0101	5.09%	0.0084	*
	Descriptive word	0.29%	0.0014	0.34%	0.0017	*
	Pronoun	14.95%	0.0315	11.74%	0.0223	***
	Numeral	3.46%	0.0072	4.17%	0.0068	***
	Quantifier	0.54%	0.0022	0.79%	0.0032	***
	Adverb	11.93%	0.0127	10.99%	0.0130	***
	Preposition	3.03%	0.0063	2.96%	0.0055	0.078
	Conjunction	4.00%	0.0084	3.39%	0.0077	***
	Auxiliary	0.28%	0.0015	0.26%	0.0012	0.205
	Exclamation	0.13%	0.0012	0.20%	0.0016	***
	Modal particle	1.60%	0.0065	1.62%	0.0064	0.655
	Onomatopoeia	0.21%	0.0033	0.77%	0.0085	***
	Set phrase	0.94%	0.0039	0.79%	0.0027	***
	Idiom	1.67%	0.0039	1.32%	0.0029	***
Emotion	Like	31.65%	0.0740	45.31%	0.0675	***
	Happy	13.11%	0.0332	16.60%	0.0404	***
	Sad	14.67%	0.0455	8.33%	0.0273	***
	Anger	0.61%	0.0066	0.54%	0.0038	0.952
	Fear	4.51%	0.0198	3.05%	0.0117	***
	Disgust	34.40%	0.0631	24.86%	0.0538	***
	Surprise	1.04%	0.0064	1.30%	0.0072	***
Personal Pronouns	First-Person Singular	6.69%	0.0028	4.16%	0.0026	***
	First-Person Plural	0.13%	0.0002	0.19%	0.0001	***
	Second-Person Singular	0.92%	0.0011	0.87%	0.0009	***
	Second-Person Plural	0.18%	0.0002	0.07%	0.0001	***
	Third-Person Singular	0.63%	0.0005	0.63%	0.0006	0.531
	Third-Person Plural	0.23%	0.0003	0.09%	0.0001	***
	Other	6.69%	0.0028	0.11%	0.0001	***
Specific Words	Negative Words	0.99%	0.0004	0.67%	0.0003	***
	Interrogative Words	2.92%	0.0006	2.18%	0.0008	***
Polarity	Negative Polarity	59.84%	0.1130	31.77%	0.1129	***
	Neutral Polarity	3.82%	0.0222	22.14%	0.1221	***
	Positive Polarity	36.33%	0.1096	46.09%	0.1190	***

Note: * *p* < 0.05, *** *p* < 0.0001.

**Table 4:** Posting habits of depression class and others.

Category	Original Weibo		Weibo with Pictures		Positioning Weibo	
	Average	Ratio	Average	Ratio	Average	Ratio
Depression class	277.42	84.21% (36,065/42,827)	51.72	18.64% (6723/36,065)	5.23	1.89% (680/36,065)
Others	561.75	61.33% (179,762/293,102)	230.07	40.96% (73,623/179,762)	33.93	6.04% (10,857/179,762)

**Table 5:** Topic analysis of depression class.

	Topic	Ratio	Keyword
1	Negative emotion fluctuation	9.41%	哈哈哈 (ha ha), 难过 (sadness), 崩溃 (breakdown), 恐惧 (fear), 压抑 (repression), 焦虑(anxiety), 抑郁 (depression), 哭泣 (crying), 疲惫 (exhaustion), 控制 (control)
2	Disease treatment and somatic responses	14.28%	吃药 (taking medicine), 医院 (hospital), 医生(doctor), 心理咨询 (psychological consultation), 头痛 (headache), 身体 (body), 呕吐 (vomiting), 恶心 (nausea), 胃痛 (stomach pain), 难受 (uncomfortable)
3	Sleep disorders	14.68%	睡不着 (sleepless), 药 (pills), 醒 (wake up), 躺(lie down), 噩梦 (nightmare), 失眠 (insomnia), 晚上 (night), 小时 (hour), 白天 (daytime), 明天 (tomorrow)
4	Sense of worthlessness	19.37%	对不起 (sorry), 废物 (waste), 垃圾 (garbage), 现实 (reality), 世界 (world), 生命 (life), 理解 (understand), 错 (wrong), 活着 (alive), 妈妈 (mom)
5	Suicidal extreme behavior	17.34%	死掉 (die), 自杀 (suicide), 活着 (live), 痛苦 (pain), 离开 (leave), 血 (blood), 割 (cutting), 刀 (knife), 打算 (plan), 念头 (thought)
6	Seek emotional support	10.70%	朋友 (friends), 陪伴 (company), 假装 (pretend), 治愈 (cure), 帮助 (help), 说话 (talk), 安慰 (console), 希望 (wish), 努力 (strive), 爱 (love)
7	Interpersonal communication	14.20%	学校 (school), 生活 (life), 工作 (work), 老师 (teacher), 小伙伴 (buddy), 父母 (parents), 住院 (hospitalization), 讨厌 (hate), 后悔 (regret), 害怕 (fear)

## Data Availability

Not applicable.
